# Comparison of Dosimetric Parameters and Clinical Outcomes in Inversely Planned Intensity-Modulated Radiotherapy (IMRT) and Field-in-Field Forward Planned IMRT for the Treatment of Breast Cancer

**DOI:** 10.7759/cureus.26692

**Published:** 2022-07-09

**Authors:** SK Azharuddin, Piyush Kumar, Navitha S, Arvind Kumar Chauhan, Pavan Kumar, Jitendra Nigam, Ankita Mehta

**Affiliations:** 1 Radiation Oncology, Shri Ram Murti Smarak Institute of Medical Sciences, Bareilly, IND; 2 Medical Physics, Shri Ram Murti Smarak Institute of Medical Sciences, Bareilly, IND; 3 Radiation Oncology, Homi Bhabha Cancer Hospital and Research Centre, Visakhapatnam, IND

**Keywords:** conformal planning, 3dcrt, imrt, breast cancer, radiation oncology, oncology

## Abstract

Introduction

Radiotherapy has been an important component of the multimodality approach to breast cancer treatment. Newer techniques like three-dimensional radiotherapy had led to better dose distribution over the target volume, with tissue inhomogeneity corrections. To improve the uniformity in dose distribution, a newer technique of intensity modulation was developed, namely, intensity-modulated radiotherapy (IMRT). The present study was designed to compare inverse planned IMRT (IP IMRT) and field-in-field forward planned IMRT (FP IMRT) in patients with breast cancer receiving post-modified radical mastectomy (MRM) adjuvant radiotherapy in terms of dosimetric parameters and clinical outcomes.

Materials and methods

Fifty patients with breast cancer who have undergone MRM and need adjuvant radiotherapy were randomly assigned in a 1:1 ratio into two groups (25 each) of IP IMRT and FP IMRT techniques. The prescribed dose was 50 Gy in 25 fractions over five weeks. In IP IMRT, five to seven tangential beams were used for the chest wall, nodal volumes were placed at suitable angles with beam optimization, and calculation was carried out by the analytical anisotropic algorithm. For FP IMRT, two opposing tangential fields were created in such a way to achieve uniform dose distribution to the planning target volume (PTV), minimizing hot spot regions, and limiting dose to the ipsilateral lung and contralateral breast. Multiple subfields were manually designed to boost the area not included in the dose cloud. The dosimetric parameters were compared for PTV, lungs, heart, left anterior descending coronary artery (LAD), opposite breast, and esophagus.

Results

The dosimetric parameters in terms of PTV are better for IP IMRT plans compared to FP IMRT plans (V95%: 92.3% vs 75.2%, p = 0.0001; D90%: 47.4 Gy vs 42.9 Gy, p = 0.0001; D95%: 44.9 Gy vs 37.1, p = 0.0004). The ipsilateral lung (V10Gy: 71.9% vs 41%, p = 0.00001; V20Gy: 42.14% vs 36.35%, p = 0.03; V40Gy: 17.31% vs 26.95%, p = 0.00004; Dmean: 20.91 Gy vs 17.88 Gy, p = 0.01) and contralateral lung (V5Gy: 31.8% vs 0.1%, p < 0.00001; V10Gy: 6.2% vs 0.08%, p = 0.0001) received statistically significant lesser doses in terms of low dose parameters in FP IMRT. In the heart, the dosimetric parameter V5 was significantly lower for FP IMRT (61.7% vs 9.7%, p = 0.00001) along with Dmean (10.92 Gy vs 4.01 Gy, p = 0.001). Similarly, LAD parameters showed comparable high dose volumes (V40Gy: 21.02% vs 16.26%; p = 0.29) in both groups and a trend toward reduction in mean dose (17.1% vs 9.2%; p = 0.05) in FP IMRT group, although low dose volumes were higher in IP IMRT group. In contralateral breast, doses in smaller volumes were better for FP IMRT plans (V0.5Gy: 59.7% vs 43.8%, p = 0.01; V0.6Gy: 54.07% vs 37.6%, p = 0.007; V1Gy: 40.9% vs 22.1%, p = 0.001; V2Gy: 28.7% vs 9.4%, p = 0.00003; V5Gy: 12.07% vs 4.2%, p = 0.0001). In esophagus, statistically significant lower doses were seen only in terms of Dmean (10.29 Gy vs 5.1 Gy; p = 0.03) with FP IMRT. No significant difference in terms of skin reactions and dysphagia was seen in both the groups.

Conclusion

Both IP IMRT and FP IMRT techniques have advantages and disadvantages, and the superiority of one technique over another cannot be established in this study. The decision for choosing one technique over another can also be based on various patient-related factors weighing the risk of loco-regional recurrences to that of manifesting radiation-induced sequelae.

## Introduction

Radiotherapy has been an important component of the multimodality approach to breast cancer treatment. Radiotherapy regimens to the whole breast after breast-conserving surgery and to the chest wall post mastectomy include conventional fractionation to a dose of 45-50 Gy in 1.8-2.0 Gy/fraction in daily fractions for five weeks or hypo-fractionated regimens of 40-42.5 Gy in 15-16 fractions, five days/week. This is followed by a boost to the tumor bed using photons or electrons to an additional 10-16 Gy in 2 Gy fractions, five days/week, in case of breast-conserving surgery. Post mastectomy, radiotherapy to the chest wall is warranted in women with advanced tumors, positive axillary lymph nodes, and inflammatory breast cancer. It helps in potentially eliminating microscopic tumor foci after surgery [1].

Conformal planning has an edge over conventional planning methods in reducing the unintentional excessive dose to normal tissues. However, the dose to surrounding organs at risk, especially the heart and lungs, is also to be considered, as it may cause serious morbidity in limiting the survival outcomes. Adjuvant nodal irradiation is usually indicated in patients with high-risk features [2]. However, there is always an increased risk of lymphedema with full axillary radiation following dissection that should be taken into account. The increasing number of long-term survivors has led to an enormous amount of literature suggesting the risk of secondary malignancies and hence careful consideration should be given in terms of dose to adjacent organs with careful follow-up of patients in the long term [3].

Latest scientific advancements led to radiotherapy techniques showing a gradual shift from conventional to conformal techniques. Newer techniques like three-dimensional radiotherapy (3DCRT) have led to better dose distribution over the target volume with tissue inhomogeneity corrections by considering variation in patient anatomy at different levels. To improve the uniformity in dose distribution, a newer technique of intensity modulation was developed, namely, intensity-modulated radiotherapy (IMRT).

Two types of planning techniques have evolved in IMRT, namely, inverse planning and forward planning. Inverse IMRT allows for a better conformal dose to the targets by allowing variation in the fluence, thus spatially modulating the intensity of the beam. Using multileaf collimators and dividing a beam into small beamlets results in fluence modification leading to a maximum dose to the target with a minimum dose to the critical organs [4]. Thus, the inverse planned IMRT (IP IMRT) technique uses tailored optimization algorithms to shape the desirable dose distributions. Compared with the IP IMRT, forward planned IMRT (FP IMRT) is a simple yet efficient and user-friendly technique with two opposing and open tangential fields placed in a way reducing the hotspot regions within the target area as well as the dose to adjacent organs at risk. Subfields are manually placed in such a way that optimal dose distribution is achieved in the dose cloud.

The present study was designed to compare IP IMRT and field-in-field FP IMRT in post-modified radical mastectomy (MRM) carcinoma breast patients receiving adjuvant radiotherapy in terms of dosimetric parameters and clinical outcomes.

## Materials and methods

A total of 50 patients with breast cancer who have undergone MRM and need adjuvant radiotherapy were selected and randomly assigned in a 1:1 ratio into two groups (25 each) of IP IMRT technique and field-in-field FP IMRT technique. Pre-treatment evaluation was done with complete clinical and physical examination, including postoperative chest wall and contralateral breast examination, baseline hematological tests, chest radiography, whole abdomen ultrasound, bone scan (if indicated), and serum alkaline phosphatase (SAP).

Patients aged >18 years with a Karnofsky Performance Scale score above 70 and no history of previous malignancy or thoracic radiotherapy were included. All such patients with prior or synchronous malignancy and distant metastasis were excluded.

CT simulation and planning

Patients were immobilized in the supine position on a semi-inclined breast board with arms extended above the head, flexed at the elbow joint, and externally rotated. Contrast-enhanced CT scans of the thorax with a slice thickness of 3 mm were obtained. Radio-opaque markers were used to mark the inferior and lateral border of the chest wall along with wires placed at the mid axillary line and surgical scar mark. After planning a CT scan, the images were acquired in Digital Imaging and Communications in Medicine (DICOM) format and were transferred to the Varian Eclipse treatment planning system (TPS) version 13.6 (Varian Medical Systems, Palo Alto, CA). Treatment plans were made based on these CT simulation datasets. Delineation of the clinical target volume (CTV), which includes chest wall and supraclavicular nodal regions along with organs at risk (OAR), was contoured according to the Radiation Therapy Oncology Group (RTOG) protocol. The planning risk volume (PRV) to the spinal cord and esophagus was taken as 5 mm and 3 mm, respectively. The planning target volume (PTV) was taken as 5 mm from CTV as per departmental protocol. Dose prescription and dose constraints were given with a target of ideal dose to PTV defined as a dose ranging from 95% to 107% relative to the prescription.

Planning technique

Inversely Planned IMRT (IP IMRT)

The IP IMRT optimized plans were generated to achieve the same objectives described for the field-in-field FP IMRT plan. A total of five to seven tangential beams were used for chest wall and nodal volumes. The beam directions were placed at suitable angles with beam optimization and calculation was carried out by the analytical anisotropic algorithm (AAA). The plans were calculated using a dynamic multileaf collimator (MLC) and jaw tracking tools.

Field-in-Field Forward Planned IMRT (FiF FP IMRT)

Two open tangential fields were created in this technique, according to the geometry defined during simulation to achieve uniform dose distribution to the PTV, minimizing hotspot regions, and limiting dose to the ipsilateral lung and contralateral breast. The tangential fields were placed in such a way that the beam entry was not passing through the opposite breast. The 95% dose cloud in a beam’s eye view projection of the treatment fields was visualized and subfields were manually designed to boost the area not included in the dose cloud. The volume receiving more than 107% was visualized and additional subfields were created to shield the region. The manual iteration of subfields was done to achieve the desired dose distribution. The number of subfields varied from one to three pairs. Either 6 or 10 MV photons were selected for the subfields depending on the separation of fields.

Dosimetric parameters

The dose constraints to the OARs were prescribed as per recommendations by the Quantitative Analyses of Normal Tissue Effects (QUANTEC) and RTOG. Dose-volume histograms (DVHs) corresponding to the delivered IMRT plans were generated for each contoured region. The dosimetric parameter under evaluation for PTV receiving 95% dose was designated as PTV V95, the dose given to 90% and 95% of the volume was designated as PTV D90% and D95%, respectively, the maximum dose to the PTV was designated as Dmax, and the mean dose to the PTV was designated as Dmean, with conformity index (CI) and homogeneity index (HI). The CI is defined as CI = TV/PTV, where TV was the volume of reference dose (95%) inside the PTV. CI value closer to 1 indicates a conformal plan. The HI is defined as HI = D2% - D98%/D50%, where D2%, D98%, and D50% are doses to the PTV volume. HI value closer to 0 indicates a homogeneous plan. To normalize the plan, the planning goal was to have a homogeneity between -5% and +7% (95-107%). The dosimetric parameters were evaluated for the ipsilateral lung (V10, V20, V30, V40, V50, Dmean, Dmax), contralateral lung (V5, V10, V20, V30, V40, Dmean, Dmax), opposite breast (V0.5, V0.6, V1, V2, V5, Dmean), spinal cord (Dmax), heart (V5, V25, V30, V40, V50, Dmean, Dmax), left anterior descending artery (LAD) (V5, V25, V30, V40, V50, Dmean, Dmax), and esophagus (V35, V50, Dmean). Daily imaging was done with kilovoltage (KV) image and once weekly with cone-beam CT (CBCT) image. Treatment delivery was done only after correcting the errors in imaging and aligning the patient to the treatment position adequately.

Clinical outcomes

Radiation toxicity (skin, contralateral breast skin, and swallowing assessment) was assessed by RTOG parameters for acute and late toxicities. Patients were assessed weekly during radiotherapy, at the end of radiotherapy, and thereafter monthly for up to six months.

Ethical considerations

The study was approved by the Institutional Ethical Review Committee of Shri Ram Murti Smarak Institute of Medical Sciences (IRB number: SRMSIMS/ECC/2018-19/216) before its inception. Written informed consent was obtained from each patient prior to participation in the study.

Statistical analysis

Collected data were analyzed using a t-test to calculate the level of significance using p-values. A p-value of < 0.05 was considered statistically significant.

## Results

A total of 50 patients of MRM who received adjuvant radiotherapy from October 2018 to March 2020 were selected and randomized into two groups. The median age of patients in the inverse IMRT group was 47 (range: 30-75 years) years and 45 years (range: 24-62 years) for the forward IMRT group. Approximately 60% of the patients in the inverse IMRT group and 36% of the patients in the forward IMRT group have had comorbidities. The most common comorbid condition was hypertension present in 18% of the entire study population (IP IMRT vs FP IMRT: 20% vs 16%) followed by diabetes mellitus (IP IMRT vs FP IMRT: 20% vs 8%) seen in another 14% of the patients.

Dosimetric parameters

Planning Target Volume

There was a significant difference in terms of parameters representing adequate dose coverage to PTV (V95, D95, D90, Dmean, and Dmax). In the inverse IMRT arm, a significantly better dose coverage is seen along with better homogeneity (p = 0.0005) and conformity index (p = 0.007) as compared to the forward IMRT arm. The dosimetric values of various parameters including PTV and OARs can be seen in Table 1.

**Table 1:** Dosimetric parameters of PTV and adjacent organs at risk IMRT: intensity-modulated radiotherapy; V%: volume in percentage; D(Gy): dose in gray; PTV: planning target volume; PRV: planning risk volume; LAD: left anterior descending artery; V95%: percentage of volume receiving 95% of the dose; V40Gy: percentage of volume receiving 40 Gy; D90%: dose received by 90% of the volume.

Parameters	Variable	Inverse IMRT	Forward IMRT	P-value
PTV	V95%	92.3	75.24	0.00001
	D90%	47.40	42.99	0.00001
	D95%	44.91	37.15	0.0004
	Dmax	54.08	54.85	0.001
	Dmean	49.68	48.18	0.00007
	Conformity index	1.1	1.2	0.0005
	Homogeneity index	0.2	0.4	0.007
Ipsilateral lung	V10	71.98	41.09	0.00001
	V20	42.14	36.35	0.03
	V30	27.86	32.35	0.05
	V40	17.31	26.95	0.00004
	V50	1.20	4.19	0.007
	Dmean	20.91	17.88	0.01
	Dmax	51.85	52.35	0.04
Contralateral lung	V5	31.85	0.18	<0.00001
	V10	6.26	0.08	0.0001
	V20	0.14	0.03	0.05
	V30	0.006	0.01	0.2
	V40	0.0002	0.003	0.1
	Dmean	3.78	0.60	0.00001
	Dmax	17.95	10.07	0.09
Opposite breast	V0.5	59.77	43.84	0.01
	V0.6	54.07	37.60	0.007
	V1	40.99	22.12	0.001
	V2	28.72	9.41	0.00003
	V5	12.07	4.20	0.0001
	Dmean	2.39	1.43	0.001
	Dmax	43.55	41.13	0.2
PRV spine	Dmax	20.74	21.002	0.4
Heart	V5	61.79	9.75	0.00001
	V25	12.88	5.15	0.02
	V30	8.77	5.017	0.09
	V40	4.26	3.75	0.4
	V50	0.16	0.21	0.37
	Dmean	10.92	4.01	0.0001
	Dmax	38.25	29.93	0.04
LAD	V5	56.57	19.52	0.018
	V25	34.96	17.54	0.06
	V30	30.54	17.23	0.1
	V40	21.02	16.26	0.29
	V50	0.19	2.93	0.08
	Dmean	17.17	9.20	0.05
	Dmax	23.58	11.09	0.08
PRV esophagus	V35	5.92	6.31	0.43
	V50	0.20	0.87	0.13
	Dmean	10.29	5.10	0.0009

Organs at Risk

In the ipsilateral lung, the low dose and medium dose regions (V10Gy, p < 0.00001; V20Gy, p = 0.03) were significantly decreased in the forward IMRT arm along with a lesser mean dose (p = 0.01). However, the high dose regions (V40Gy, p = 0.00004; V50Gy, p = 0.007) were significantly lesser in the inverse IMRT arm as compared to the forward IMRT arm, whereas in the contralateral lung, the low dose parameters (V5Gy, p < 0.00001; V10Gy, p = 0.001) were significantly lesser in the forward IMRT arm as compared to the inverse IMRT arm with no significant difference observed in medium and high dose regions of V20, V30, and V40. The low dose parameters in the opposite breast (V0.5Gy, p = 0.01; V0.6Gy, p = 0.007; V1Gy, p = 0.001; V2Gy, p = 0.00003; V5Gy, p = 0.0001) were significantly lesser in the forward IMRT arm as compared to the inverse IMRT arm along with mean dose (p = 0.001). No significant difference was observed in Dmax in both the techniques with respect to the PRV spine. The low dose regions (V5, p < 0.00001) were significantly lesser in the forward IMRT arm in the heart and LAD. There was no significant difference observed in the two techniques in terms of high dose regions. However, the maximum dose received to LAD was significantly higher (p = 0.08) in the inverse IMRT arm in comparison with the forward IMRT arm. The mean dose in the PRV esophagus was significantly lesser in the forward IMRT arm (p = 0.0009) with no difference in terms of high dose regions.

Radiotherapy reactions

The incidence of radiotherapy reactions is quantified in Table 2. The radiation-induced skin reactions were slightly higher in the forward IMRT group than in the inverse IMRT group (48% vs 56%), but the result was non-significant (p = 0.29). The percentage of patients with esophageal reactions was slightly higher in the inverse IMRT group as compared to the forward IMRT group (28% vs 16%), but the result was non-significant (p = 0.15).

**Table 2:** Total percentage of patients showing radiotherapy reactions in both the arms IMRT: intensity-modulated radiotherapy.

Parameters	Inverse IMRT	Forward IMRT	P-value
Skin reaction (dermatitis)	48%	56%	0.29
Esophageal reactions (dysphagia)	28%	16%	0.15

## Discussion

Adjuvant breast irradiation after mastectomy is considered the standard of care, resulting in a significant reduction of loco-regional recurrences. Regarding radiotherapy techniques, the tangential field technique is the most common modality in most centers. However, many centers use IP IMRT, owing to specific advantages in terms of planning, although no significant clinical advantage is appreciated in terms of risk of recurrence or survival over tangential field techniques. The tangential field technique had several drawbacks, including the lack of conformal and steep dose gradient beyond the target volume.

Various studies have suggested that field-in-field (FiF) IMRT potentially leads to an acceptable dose distribution to the target volumes as well as achieving a maximum predefined tolerance limit to the OARs compared with the inverse IMRT group. IP IMRT can improve the target dose coverage by permitting each beam of radiation to be custom-tailored according to the specific geometrical shape of the breast tumor. IMRT has proved to be advantageous particularly because of homogenous dose distribution of the target area with the use of MLC, modulating fluence, and dividing a beam into small beamlets to prescribe maximum dose to the target with minimum dose to the critical organs. The major concerns lie in the increased risk of long-term cardiopulmonary complications and second malignancies. The lung and heart remain the two most critical organs stressed in breast malignancies. The beneficial role and superiority of either of the techniques in post-mastectomy breast cancer radiotherapy are controversial in the existing literature.

Dosimetric parameters

In our study, the target dose coverage of PTV was significantly better in the IP IMRT group than in the FP IMRT group in terms of dosimetric parameters, which include V95% (92.3% vs 75.24%, p = 0.00001), D95% (44.91 Gy vs 37.15 Gy, p = 0.0004), D90% (47.4 Gy vs 42.9 Gy, p = 0.00001), Dmean (49.68 Gy vs 48.18 Gy, p = 0.00007), and Dmax (54.08 Gy vs 54.85 Gy, p = 0.001). CI (p = 0.007) and HI (p = 0.0005) were also better in the inverse IMRT group. This is because of the multi-beam arrangement in IP IMRT, which ensured adequate build-up thickness in all directions allowing better coverage of PTV. A study by Al-Rahbi et al. compared the dosimetry of 20 patients with IP IMRT plans and FiF forward plans in breast cancer patients. In terms of coverage of the PTV, optimal dose distribution was achieved with both techniques with 95% of the volume receiving more than 95% of the prescribed dose. But FP IMRT plans were significantly better in terms of V95% (95.5% vs 97.5%, p = 0.01), V90% (96.6% vs 97.5%, p = 0.01), and Dmean_ _(99.% vs 100.7%, p = 0.02). The Dmax was also significantly lower in the FP IMRT group compared to the IP IMRT group (111% vs 107%, p = 0.01), with statistically significant values. No significant difference was observed in HI in both the plans but the CI was significantly better in the IP IMRT group than in the FP IMRT group (0.76 vs 0.57, p = 0.01) [5].

In another study by Fontanilla et al. published in 2012, a retrospective analysis was done on 20 post-mastectomy patients receiving radiotherapy by the forward planning conventional technique. This study analyzed that the dose to the chest wall was 74% (V45Gy = 74%). It was concluded in this study that excellent local control was achieved even with these dose targets on long-term follow-up [6]. Similar results were observed in our patients who underwent forward planning where the mean dose of V95% to the chest wall was 75.24%. The dose to normal adjacent structures was minimal in comparison to the inverse planning technique. There is a wide variation among various studies in terms of superiority of coverage of PTV between both planning techniques. It is imperative that the difference in terms of adequate dose to the PTV can be explained by the variation in the number, orientation, and weightage of beams along with different iterative objectives and the priority defined in relation to the various organs at risk in IP IMRT technique. The problem encountered in FP IMRT plans was suboptimal coverage of nodal volumes, superficial build-up region of the chest wall, and the junctional dose.

The two opposing tangential beams from either side of the chest wall in FiF forward IMRT plans led to heterogeneous dose distribution. The use of a single anterior supraclavicular field with a dose prescription at the maximum depth in forward IMRT plans led to inadequate and in-homogeneous coverage of nodal volumes with in-homogeneous dose distribution. The delineated region of the supraclavicular region also varied enormously with the patient’s anatomy and the routine use of prescription at maximum depth did not optimally cover intended targets for all the patients, while it resulted in overdosage to the lung tissue in a proportion of patients. A higher number of beams in inverse IMRT plans ensured better and homogenous coverage of the PTV as the multi-beam arrangement ensured adequate build-up thickness across all directions and tangential beams were utilized for nodal volumes as they led to optimal coverage. The beam optimization allowed steep dose fall-off and better conformity.

In a dosimetric study by Karacetin et al. comparing 3DCRT, IP IMRT, and FiF FP IMRT groups in left-sided breast cancer patients, the low dose volumes of ipsilateral lungs, i.e., V5Gy doses (62.4% vs 28.2%, p = 0.001) and V10Gy doses (30.3% vs 20.18%, p < 0.03), in inverse IMRT plans were significantly higher compared with forward IMRT plans. A significant difference was also observed in with higher doses in inverse IMRT plans [7]. This is similar to our study demonstrating a significant raise in low dose volumes with the IP IMRT technique in terms of V10Gy (71.98% vs 41.09%, p < 0.00001). Although there was no significant difference in V20Gy doses in their study, in our study, V20Gy (42.14 vs 36.35, p = 0.03) was also significantly higher in the inverse IMRT arm. The possible explanation being a higher number of beams (up to seven) were utilized in our plans compared to the five-beam arrangement in their study. Our study proved that FP IMRT technique is significantly better in terms of low dose and medium dose regions, i.e., V10Gy (71.98% vs 41.09%, p < 0.00001) and V20Gy (42.14 vs 36.35, p = 0.03), along with a lesser mean dose (20.91 Gy vs 17.88 Gy, p = 0.01). However, the high dose volumes because of better conformity, i.e., V30Gy (27.86% vs 32.35%, p = 0.05), V40Gy (17.31% vs 26.95%, p = 0.00004), and V50Gy (1.20% vs 4.19%, p = 0.007), were significantly lesser in the inverse IMRT arm as compared to the FP IMRT arm.

A single supraclavicular field in forward IMRT plans led to more exposure of the apex of the lung to the prescribed doses. The superficial lung tissue beneath the chest wall was also largely exposed to high doses. However, the decrease in low dose volumes was large and may translate into a decreased risk of long-term pulmonary complications with forward IMRT plans but this requires clinical validation with long-term follow-up.

With respect to the contralateral lung, there was a statistically significant difference in terms of low dose regions and Dmean favoring FiF forward IMRT plans. The dosimetric parameters including V5Gy (31.85% vs 0.18%, p < 0.00001) and V10Gy (6.26% vs 0.08%, p = 0.0001) along with Dmean (3.78Gy vs 0.60Gy, p = 0.0001) were significantly better in forward planning group with comparable results in high dose regions (V30 and V40). The possible explanation regarding the lower doses of the contralateral lung with the FiF FP IMRT technique lies in the direction of the opposing tangential fields chosen in such a way that the entry dose through the contralateral lung was kept minimum. Similar results were observed in a population-based comparison study of normal lung doses and radiation pneumonitis done by Li et al. in post-mastectomy patients, proving that the V5Gy of the contralateral lung was significantly lower in the two tangential field plans (14% ± 7%) than in the inverse planning IMRT group (12% ± 4%, p = 0.02). However, no statistically significant difference was seen between the two techniques in terms of Dmean [8].

A dosimetric study by Al-Rahbi et al. proposed various dosimetric parameters of contralateral breast dose-volume thresholds that include V0.5Gy, V0.6Gy, and V2.0Gy, and the mean dose that serves as the dose objective in treatment planning and were used to monitor the dose distribution to opposite breast, thereby limiting the risk of second malignancy. The low dose volumes V0.6Gy (p = 0.07), V1Gy (p = 0.03), V2Gy (p < 0.00001), and V5Gy (p = 0.02) were significantly less in the FiF FP IMRT technique than in the inverse IMRT technique. There was also a significant benefit in terms of Dmean (p < 0.00001) in FP IMRT plans thus concluding that there was a significant benefit in low dose volumetrics as well as mean doses in the FiF FP IMRT arm [5]. Our study had similar findings with inverse IMRT demonstrating a significant rise in the mean dose (2.39 Gy vs 1.43 Gy, p = 0.001) and low dose volumes (V0.5Gy: 59.77% vs 43.84%, p = 0.01; V0.6Gy: 54.07% vs 37.60%, p = 0.007; V1Gy: 40.99% vs 22.12%, p = 0.001; V2Gy: 28.72% vs 9.41%, p = 0.00003; V5Gy: 12.07% vs 4.20%, p = 0.0001) of opposite breast. Hence, inverse IMRT plans are particularly gaining concern for the development of secondary malignancies in long-term survivors.

No significant difference in the maximum dose of PRV of the spinal cord (p = 0.1) was noted. There is a limited concern across the literature regarding dose to the spinal cord or any risk of myelopathy in breast cancer irradiation.

Dose to the heart is a concern in breast cancer patients. It has been well documented in the literature that standard conventional techniques of opposing tangential radiotherapy fields used for breast cancer may result in increased toxicity in cardiac tissues and that incidental dose to the heart results in adverse events such as diffuse myocardial fibrosis, pericarditis, coronary artery disease (CAD), and in some rare circumstances, valvular heart disease [9]. With improvement in technology and treatment planning, better sparing of normal tissues was achieved along with a decrease in toxicity profile, although breast cancer-related cardiac toxicity typically takes a decade or more years to manifest [10]. The dose to LAD has been proven to be an important predictor of coronary events and cardiac deaths [11]. Al-Rahbi et al. demonstrated that the high dose volume, i.e., V30Gy for the heart, was found to be comparable in both the forward IMRT plan as well as the inverse IMRT plan while the mean dose of the heart was significantly lesser for the forward IMRT compared to the inverse IMRT [5]. Our study also showed similar findings with comparable high dose volumes amongst both the techniques and a huge significant reduction in mean dose (16.6 Gy vs 5.2 Gy, p < 0.00001) in the forward IMRT plans. However, low dose volumes were higher in IP IMRT owing to its multi-beam arrangements. Better results with forward planning may be explained by the angle of the two tangential beams that were chosen to maximally avoid the entry dose through the LAD.

With respect to the esophagus, there was a statistically significant reduction in terms of Dmean (10.29 Gy vs 5.1 Gy, p = 0.03) with FP IMRT. No statistical significance was seen in high dose parameters like V35Gy (5.92% vs 6.31%) and V50Gy (0.20% vs 0.87%). However, it was observed that all the parameters were in the acceptable range as defined by QUANTEC (mean dose < 34 Gy, V35 < 50%, V50 < 33%). Although the risk of second malignancies of the esophagus in long-term survivors of breast cancer patients is a matter of concern because of significantly higher mean doses in the inverse IMRT technique (>10 Gy), longer follow-up is needed for further evaluation and validation.

Clinical outcomes

The common radiation-induced acute reactions are dermatitis and esophagitis. These reactions may vary from mild reactions including erythema and low-grade dysphagia to severe reactions like fibrosis and stricture.

It has been observed that most patients present with these skin reactions in the second to the third week of radiotherapy reaching their peak toward the end of treatment. A study by Phansopkar et al. on 54 postoperative patients including both MRM and breast-conserving surgery (BCS) showed onset of reactions in patients opposing tangential field plans within the first week as compared to onset in the third week in the inverse IMRT group. In the fifth week during the end of treatment, the grade III reactions were seen in 19.14% of opposing tangential field patients as compared to 28.57% in the inverse IMRT group [12]. Our study showed comparable results in both the groups with no significant difference (p = 0.29), but with a slightly lower number of patients, i.e., 48% in the inverse IMRT group developing skin reactions as compared to 56% in the FiF forward group.

A study by Lamart et al. observed that supraclavicular nodal irradiation is the most important factor determining the dose to the esophagus with comparatively lower doses from chest wall tangential and axillary fields [13]. Emami et al. postulated that the percentage of patients with late complications including esophageal stricture and perforation was only 5% even in those receiving a dose of 60 Gy to 1/3 of volume [14]. However, clinical trials of RTOG recommend keeping the mean dose to less than 34 Gy to minimize acute esophagitis [15]. In our study also, the maximum number of patients received supraclavicular irradiation. The percentage of patients with reaction was slightly higher in the FP IMRT group than in the inverse IMRT group (28% vs 16%), but the result was non-significant (p = 0.29).

In the present study, the inverse IMRT group proved to be significantly better in PTV coverage. The dose in-homogeneity was minimized and a sharp dose fall-off beyond the target volume was seen. The better conformity translated into a reduction in high dose volumes of the ipsilateral lung. But the accompanying disadvantage was a rise in the low dose volumes and mean dose of surrounding OARs. The LAD is recognized as an important predictor of radiation-induced coronary events but the impact of the technique on its dosimetry has not been much stressed. The present study showed a significant reduction in low dose volumes with a trend toward significant reduction even in high dose volumes in the FP IMRT group. This is particularly gaining concern because of the increasing number of long-term survivors, which may translate in the long term into an increased cardiac morbidity and mortality, chronic pulmonary sequelae, and a higher incidence of second malignancies and impact the advantage of radiation. At the same time, better dosimetry of PTV might also translate to a better long-term loco-regional control offered by radiation. But long-term data and a larger study population are required to validate the findings.

Limitations

The major limitation of our study is that normally patients with breast cancer who are been treated by radiotherapy should be under DIBH (deep inspiration breath hold) protocol to get better sparing of the heart, which needs a lot of training for patients as well as the technical staff and is also difficult to execute on reality grounds. The present study could have incorporated the DIBH protocol, which may have led to better dosimetric parameters for heart and LAD. Also, the patients selected and randomly assigned into two groups may have introduced bias in the study owing to the differences in patients' anatomy along with CTV parameters like the inclusion of supraclavicular nodal volumes, which can be excluded by planning both the techniques in the same patient; however, this was not the objective of our study. Further, the number of patients was limited due to time-bound research activity and more patients may have helped to validate the current findings.

## Conclusions

A clear advantage or superiority of one technique over another cannot be established in this study. The decision for choosing one technique over another can also be based on the various patient-related factors weighing the risk of loco-regional recurrences to that of manifesting radiation-induced sequelae. The drawback of the forward IMRT group in terms of PTV coverage might be dealt with by optimizing the beam angles, beam energy, and number and weightage of individual subfields.
